# Cultivation of lipid-producing microalgae in struvite-precipitated liquid digestate for biodiesel production

**DOI:** 10.1186/s13068-018-1102-3

**Published:** 2018-04-07

**Authors:** Yiqi Jiang, Xiaodong Pu, Dan Zheng, Tao Zhu, Shuang Wang, Liangwei Deng, Wenguo Wang

**Affiliations:** 1 Biogas Institute of Ministry of Agriculture, Chengdu, 610041 People’s Republic of China; 2 Key Laboratory of Development and Application of Rural Renewable Energy, Chengdu, 610041 People’s Republic of China

**Keywords:** Liquid digestate, Struvite precipitation, Microalgae, *Dictyosphaerium ehrenbergianum*, Biodiesel

## Abstract

**Background:**

Using liquid digestate from the biogas industry as a medium to culture lipid-producing microalgae is considered mutually beneficial for digestate valorization and for reducing the cost of microalgal cultivation. However, the low transmittance and high ammonium (NH_4_^+^-N) levels in liquid digestate negatively influence microalgae growth.

**Results:**

Struvite precipitation was used to pretreat liquid digestate. To obtain struvite-precipitated supernatant with an ideal transmittance, NH_4_^+^-N concentration, salinity, and N:P ratio for microalgal growth, there should be a 1:1.2:1.2 NH_4_^+^:Mg^2+^:PO_4_^3−^ molar ratio in the liquid digestate, with KH_2_PO_4_ and MgCl_2_ added through continuous stirring. The addition and stirring was subsequently stopped when the pH reached 8.5. Of the nine tested microalgae species, *Dictyosphaerium ehrenbergianum* exhibited the best growth in the supernatant. The biomass productivity and lipid content of *D. ehrenbergianum* cultured in the struvite-precipitated supernatant were 161.06 mg/l/days and 34.33%, respectively, which was higher than when cultured in the standard BG-11 medium. Moreover, the struvite-precipitated supernatant improved the accumulation of monounsaturated fatty acids and saturated fatty acids.

**Conclusions:**

This study described a new way to combine liquid digestate treatment and microalgal biodiesel production. The struvite-pretreated liquid digestate can be used to culture *D. ehrenbergianum* for biodiesel production.
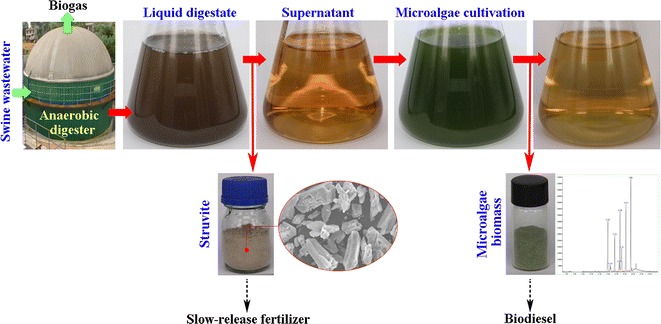

## Background

Microalgae are considered one of the most promising biofuel feedstocks [1, 2]. However, the high cultivation cost is still a limiting factor for its further commercialization [3]. Using wastewater for microalgal cultures is considered mutually beneficial, because the wastewater provides nutrients for microalgal growth, while the microalgae remove pollutants from the wastewater [4, 5].

Digestate is an important byproduct of the anaerobic digestion of organic waste [5, 6]. Traditionally, land application is the primary method for managing digestate from livestock farms [7]. However, with the rapid development of the biogas industry, the volume of digestate has increased substantially in recent years. Land application and other digestate processing techniques require large energy inputs and increase environmental risk, especially with respect to liquid digestate from large scale biogas plants in large livestock or poultry farms [6]. Digestate management has become a major bottleneck in the development of the biogas industry, as well as for the livestock and poultry breeding industry [8]. On the other hand, the liquid digestate is rich in nitrogen (N), phosphorous (P), potassium (K), and other nutrients essential for microalgal growth [9]; thus, its use as a microalgal culture medium is considered a new opportunity for digestate valorization [6, 9].

Culturing microalgae in liquid digestate can reduce the cost of nutrients for microalgal cultivation, while simultaneously reusing liquid digestate [9]; however, there are some limiting and inhibitory factors in liquid digestate-based microalgal cultivation. First, the high turbidity of liquid digestate, caused by suspended materials, can lead to low transmittance. This reduces the efficiency of photosynthesis and the growth of microalgae [10]. Moreover, the ammonium-nitrogen (NH_4_^+^-N) levels in liquid digestate are usually high and can potentially inhibit microalgal growth [11]. Pretreatments, such as separation and dilution, are often used to reduce the negative effects associated with high NH_4_^+^-N and turbidity; however, these processes consume a large quantity of energy and fresh water [9].

Struvite (magnesium ammonium phosphate; MAP) precipitation can reduce NH_4_^+^ and suspended solids (SS) in wastewater under alkaline conditions, while simultaneously generating slow-release fertilizer [12, 13]. This technique has been also used to recover N from digestate [14]. However, the pH of liquid digestate is usually neutral or weak alkaline, and it has less PO_4_^3−^ and Mg^2+^ compared to NH_4_^+^. As such, chemicals containing PO_4_^3−^, Mg^2+^, or OH^−^ must be added to facilitate effective NH_4_^+^ removal [15, 16]. As a result, the candidate supernatant for microalgal culture following precipitation usually has a high pH and salinity, as well as an unsatisfactory N:P ratio for microalgal growth. The suggested optimal N:P mass ratio for microalgal growth is approximately 7:1, based on the composition of microalgae [17]; however, the N:P molar ratio in liquid digestate is usually adjusted to approximately 1:1 (mass ratio around 0.45) at the beginning of the reaction to achieve a high precipitation efficiency [18, 19]. In fact, the remaining N and P levels, as well as the N:P ratio in the supernatant, are under the control of the reaction conditions, such as pH value and PO_4_^3−^:Mg^2+^:NH_4_^+^ molar ratios [19]. Thus, one goal of this study was to determine the optimal combination of chemical additives and reaction conditions of struvite precipitation in liquid digestate, to obtain an optimal supernatant for microalgal growth.

Most microalgae prefer neutral environments; as such, the high pH and salinity in the struvite-precipitated supernatant may negatively affect algal growth. However, reducing the pH and salinity in the supernatant is usually not economical. Microalgal species have a range of optimal pH and salinities, with some species tolerating high pH and salt conditions [20]. Selecting high pH and salt tolerant species is beneficial for culturing microalgae in liquid digestate after struvite precipitation. Therefore, in this study, struvite precipitation was selected as a digestate pretreatment technique to determine the optimal combination of chemical additives and reaction conditions, and to investigate suitable microalgal species. This provides a new way for combining liquid digestate treatment and microalgal cultivation [21].

## Methods

### Liquid digestate collection and characterization

The liquid digestate used in this study was obtained from a pig farm located in Jianyang, Sichuan province in China. The samples were collected from a storage pond after treatment in an anaerobic continuous stirred tank reactor (CSTR) with the raw materials of swine wastewater after separation. They were immediately transported to the laboratory and stored at 4 °C until use.

### Microalgal strains and growth medium

Nine microalgal strains were collected from the Freshwater Algae Culture Collection at the Institute of Hydrobiology (FACHB-Collection, Wuhan, China), including *Chlorella regularis* FACHB-1068, *Chlorella pyrenoidosa* FACHB-9, *Botryococcus braunii* FACHB-357, *Scenedesmus obliquus* FACHB-417, *Dictyosphaerium ehrenbergianum* FACHB-1223, *Haematococcus pluvialis* FACHB-712, *Spirulina subsalsa* FACHB-351, *Spirulina platensis* FACHB-900, *and Spirulina maxima* FACHB-438. The three *Spirulina* strains were cultured in the Spirulina medium [22] and the other strains were cultured in BG-11 medium [23].

### Experimental procedures

#### Struvite precipitation

**Fig. 1:**
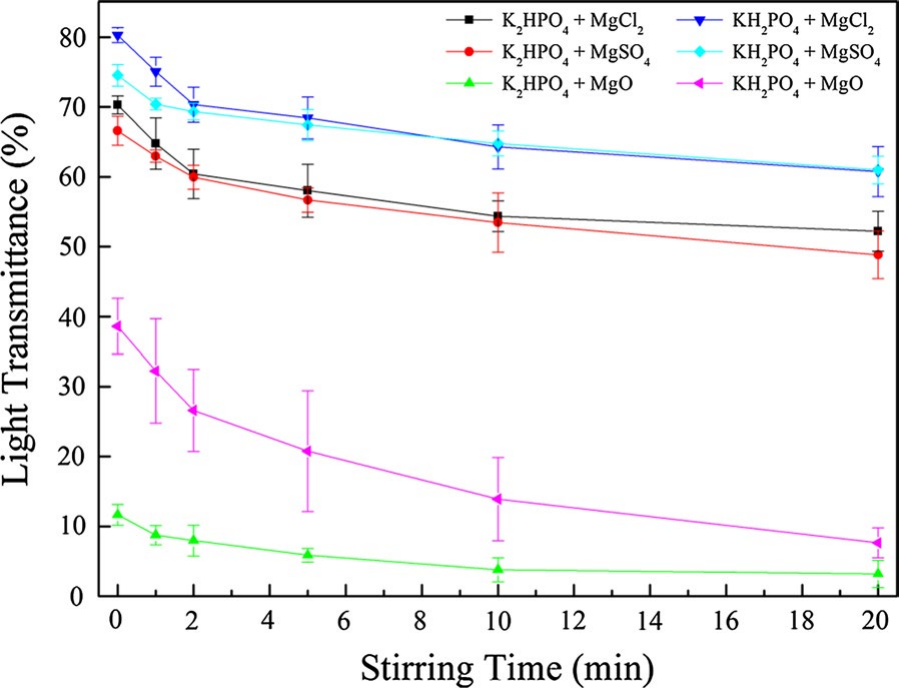
Effect of Mg^2+^ and PO_4_^3−^ sources and stirring time on transmittance (NH_4_^+^:Mg^2+^:PO_4_^3−^ molar ratios at 1:1.2:1.2, pH at 9.0, and constant rate at 150 rpm). Errors bars show standard deviation (*n* = 3)

MgSO_4_·7H_2_O, MgCl_2_·6H_2_O, and MgO were used as sources of Mg^2+^; and K_2_HPO_4_·3H_2_O, KH_2_PO_4_, and NaH_2_PO_4_ were used as sources of PO_4_^3−^; Fig. 1 and Table 1 show the combinations tested in this study.

**Table 1:** Effect of KH_2_PO_4_ and NaH_2_PO_4_ on transmittance, NH_4_^+^-N removal rate, and salinity

Combinations	Transmittance (%)	Salinity (%)	NH_4_^+^-N removal rate (%)
KH_2_PO_4_ + MgCl_2_	80.83 ± 0.43^a^	0.67 ± 0.03^a^	93.18 ± 0.74^a^
NaH_2_PO_4_ + MgCl_2_	74.76 ± 0.11^b^	0.77 ± 0.05^b^	89.18 ± 0.02^b^

Results are expressed as the mean ± SD (*n* = 3)

^a,b,c^Different letters in the same row indicate significant differences at *p* < 0.05

To evaluate the effect of the Mg^2+^ and PO_4_^3−^ sources and stirring time on struvite reaction, 300 ml of liquid digestate were fed into a 500-ml beaker. After this, different combinations of Mg^2+^ and PO_4_^3−^ sources were added and mixed by continuous stirring with a magnetic stirrer at a constant rate (150 rpm) to make the PO_4_^3−^:Mg^2+^:NH_4_^+^ molar ratios of each solution at 1:1.2:1.2. When the exogenous compounds were dissolved, the pH of the mixture was adjusted to 9.0 using 0.1-M NaOH [19]. The stirring continued for 0, 1, 2, 5, 10, and 20 min after the pH reached 9.0; stirring then stopped to allow sampling. A 10-ml sample of each mixture was placed into its own 15-ml centrifuge tube. After settling for 30 min, the supernatant from each centrifuge tube was used for analysis.

To select the best precipitation conditions for optimal microalgal growth in terms of residual NH_4_^+^-N concentration and N:P mass ratio, further experiments were done at different pH values (8.0, 8.5, and 9.0) and at different P:Mg:N molar ratios (1:1:0.95, 1:1:1, and 1:1.2:1.2) with the ideal Mg^2+^ and PO_4_^3−^ sources and stirring pattern identified above.

#### Microalgal cultivation

The struvite-precipitated supernatant obtained above acted as a nutrient source to cultivate nine microalgal strains. Each strain was cultivated in an Erlenmeyer flask (250 ml) as a single batch (100-ml working volume) at a constant temperature (25 °C). A 12-h light/12-h dark cycle was provided using daylight fluorescent tubes with a photon flux density of 40–50 μmol/m^2^/s. The cultures were manually shaken 2–3 times per day to prevent biomass sedimentation as described previously [24]. The biomass in each Erlenmeyer flask was after 7-day cultivation.

Once a suitable microalgal strain was chosen, laboratory-scale cultivations were conducted in 1.2 l reactors (1 l working volume) for 10-day cultivation. Either struvite-precipitated supernatant or BG-11 was used as the culture medium. An ambient air flow of 0.2 l/min was provided to each reactor in a 12 h/12 h cycle, consistent with the photoperiod described above.

### Analytical methods

#### Water quality analysis

The pH value and salinity of the solution was measured using a pH meter and conductivity meter, respectively. Transmittance was measured using spectrophotometry at 680 nm [25]. Chemical oxygen demand (COD) was determined according to standard methods described by APHA [26]. After filtration through a 0.45 μm membrane, the concentration of NH_4_^+^-N and NO_3_^−^-N was analyzed using an AA-3 autoanalyzer (Bran + Luebbe, Germany). PO_4_^3−^-P was measured by the molybdate-ascorbic acid colorimetric method described by APHA [26]. Scanning electron microscopy and X-ray diffraction analysis were used to analyze the struvite precipitate.

The struvite precipitate collected from the bottom of the beakers was dried at room temperature (25 ± 2 °C) and ground using a mortar. The powder (with particles that passed through a 200 mesh) was imaged using scanning electron microscopy (Hitachi SU1510, Japan) with an energy-dispersive spectrometer system (SEM–EDS) (Horiba EX-250, Japan). The crystal structures of struvite precipitation were measured using X-ray powder diffractometer (XRD) (Bruker D8 ADVANCE, Germany). The scattering was operated at a power level of 60 kV and at 80 mA. The data were recorded at a speed of 4°/min over the angular range of 10°–60°.

#### Microalgal growth and total lipid analysis

The specific growth rate (μ) based on dry cell weight (DCW) was used to evaluate the growth of each microalgal strain in the struvite-precipitated supernatant. To determine DCW, 10-ml microalgae samples were collected and centrifuged at 3200*g* for 10 min and then washed twice in 0.5 M of ammonium formate (HCOONH_4_) to remove impurities. The harvested microalgae were dried in an oven at 60 °C until the samples reached a constant weight. The samples were subsequently cooled to room temperature in a desiccator before weighing. The specific growth rate (μ) was calculated as follows:

1 $$\upmu = ({ \ln }X_{\text{final}} {-}{ \ln }X_{0}) /(t_{\text{final}} {-}t_{0} ).$$

In this expression, *X*_final_ and *X*_0_ are DCW (mg/l) at the first (*t*_0_) and last time point (*t*_final_), respectively.

Growth curves based on DCW, biomass productivity (BP), and lipid productivity (LP) were used to calculate the growth status of the selected microalgal strain. To draw the growth curve, 10-ml microalgae samples were collected from the 1.2-l reactors every day, and the DCW was measured as described above. At the end of the exponential phase, the BP was calculated using the following equation:

2 $${\text{BP}}\left( {{\text{mg}}/{\text{l}}/{\text{day}}} \right) = \left( {{\text{DCW}}t_{\text{final}} {-}{\text{DCW}}t_{0} } \right)/\left( {t_{\text{final}} {-}t_{0} } \right).$$

This calculation applied two time intervals of DCW (mg/l).

The total lipid content was extracted using an extraction method adapted from Bligh [27]. Approximately 0.1 g of dried microalgae powder was transferred into a 10-ml glass tube, and 3-ml 2:1 chloroform–methanol (v/v) mixture was then added. This mixture was blended with a vortex mixer at room temperature for 10 min. Then, 1-ml methanol was added to the mixture and blended for 1 min; 1.8-ml distilled water was then added to the mixture and blended for 5 min. Finally, the mixture was centrifuged at 3200*g* for 10 min. The organic phase was transferred to another glass tube and washed twice using a 5% NaCl solution. The final organic phase was carefully collected, the solvents were dried at 60 °C in an oven, and the weight of the remaining lipids was recorded. The total lipid content was calculated as a percentage of the total biomass (in % DCW). LP was calculated using Eq. (3):

3 $${\text{LP}}\left( {{\text{mg lipid}}/{\text{l}}/{\text{day}}} \right) = {\text{BP}} \times {\text{lipid content}} \times 100.$$

#### Fatty acid profile analysis

Fatty acid content and composition analysis were determined in two consecutive steps, including the preparation of fatty acid methyl ester (FAME) and the analysis using Gas Chromatography–Mass Spectrometry (GC–MS) (Agilent, USA). FAME was prepared using a one-step extraction–transesterification method, as described by Indarti et al. [28], with a minor modification. Dried microalgal samples (approximately 500 mg) were weighed into clean, 50-ml screw-top glass bottles, to which a 20 ml mixture of methanol, concentrated sulfuric acid, and chloroform (4.25:0.75:5) were added. Transesterification was carried out in a 90 °C water bath for 90 min. Once the reaction was completed, the chloroform layer containing the FAME was carefully collected for GC–MS analysis. The oven temperature was set at 80 °C, held steady for 5 min, was then raised to 290 °C at a rate of 4 °C/min, and was then held at 290 °C for 5 min. The resulting compounds were identified in the NIST Mass Spectral Database and quantified by the area normalization method.

### Statistical analysis

Each experiment was performed in triplicate and was repeated at least three times. The experimental results were reported as the mean value of each parameter with standard deviation. Statistical analysis was performed using a one-way analysis of variance (ANOVA) followed by a Tukey pairwise comparison, using Origin 10.0. A *p* value < 0.05 was considered statistically significant.

## Results and discussion

### Optimization of struvite precipitation

The concentrations of COD, NH_4_^+^-N, NO_3_^−^-N, and PO_4_^3−^-P in the digestate used in this study were 629.05, 591.2, 0.07, and 9.87 mg/l, respectively. The pH value was 8.2; the transmittance was 0.11%; and the salinity was 0.26%.

Additional Mg^2+^ and PO_4_^3−^ ions were required to achieve high struvite precipitation efficiency in the liquid digestate. The sources of Mg^2+^ and PO_4_^3−^ significantly affected the quality of the struvite-precipitated supernatant [12]. In this study, six combinations were first tested at a NH_4_^+^:Mg^2+^:PO_4_^3−^ molar ratio of 1:1.2:1.2 and a pH of 9.0, as described by Perera et al. [19]. Figure 1 shows that the combination of KH_2_PO_4_ + MgCl_2_ resulted in the highest transmittance at the first test time point (0 min), reaching 80%; this combination was followed by KH_2_PO_4_ + MgSO_4_, K_2_HPO_4_ + MgCl_2_, and K_2_HPO_4_ + MgSO_4_, which resulted in transmittances of more than 60%. The two MgO combinations exhibited low transmittance due to the low solubility, while the K_2_HPO_4_ + MgO combination resulted in the lowest transmittance of approximately 10%. This indicated that KH_2_PO_4_ and MgCl_2_ were the better choices for obtaining supernatant with high transmittance after struvite precipitation.

In addition, the maximum transmittance of each combination occurred when the pH reached 9.0, and it gradually decreased with continued stirring (Fig. 1). The struvite formed flocs and rapid precipitation reduced the suspended solids, thereby improving the transmittance of the liquid digestate. Continuous stirring could destroy the flocs, causing suspended fine particles that could decrease the transmittance. Therefore, in subsequent experiments, the stirring was stopped as soon as the pH reached the predetermined value.

**Fig. 2:**
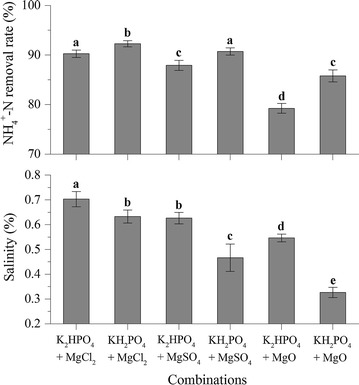
Effect of Mg^2+^ and PO_4_^3−^ sources on NH_4_^+^-N removal rate and salinity (NH_4_^+^:Mg^2+^:PO_4_^3−^ molar ratios at 1:1.2:1.2, the stirring was stopped as soon as the pH reached to 9.0). Errors bars show standard deviation (*n* = 3). Different letters indicate a significant difference at *p* < 0.05

The NH_4_^+^-N removal rate and the salinity of the supernatant were measured after 30 min of sedimentation at the first test time point (0 min). The NH_4_^+^-N removal rate displayed the same trends as the transmittance (Fig. 2). The combination of KH_2_PO_4_ + MgCl_2_ also resulted in the highest NH_4_^+^-N removal rate, at more than 90%. In addition, the KH_2_PO_4_ resulted in a lower salinity, while the MgCl_2_ resulted in a higher salinity than with MgO and MgSO_4_; however, the two MgO combinations were not considered due to low light transmittance and NH_4_^+^ removal. The remaining two KH_2_PO_4_ combinations were considered as alternatives. The salinity of KH_2_PO_4_ + MgCl_2_ was 0.63%, which was slightly higher than KH_2_PO_4_ + MgSO_4_.

In nature, microalgae have a large species diversity, allowing them to adapt to salinity changes. In the open ocean, salinity varies between 3.3 and 3.7% [29], which is significantly higher than the salinity obtained in this study. Moreover, salt stress could increase the lipid content of some microalgal species [30]. Of the six combinations, we selected KH_2_PO_4_ + MgCl_2_ for further investigation. Other studies have confirmed that the dominant form of P in MAP formation reaction is H_2_PO_4_^−^ or HPO_4_^2−^ [31]. In this study, the H_2_PO_4_^−^ achieved better results with higher transmittance, NH_4_^+^-N removal rate, and lower salinity. Yetilmezsoy and Sapci-Zengin [32] found similar results when recovering NH_4_^+^-N from the effluent of a UASB treating poultry manure using MAP precipitation.

NaH_2_PO_4_ is often used as a PO_4_^3−^ source for struvite precipitation [12]. As such, we further analyzed the differences between NaH_2_PO_4_ and KH_2_PO_4_. The results indicate that the KH_2_PO_4_ + MgCl_2_ combination achieves higher transmittance, higher NH_4_^+^ removal rate, and lower salinity (Table 1). The MgKPO_4_·6H_2_O (MKP) is one of the struvite analogs, but it is more likely to form if NH_4_^+^ concentrations are low [31, 33]. The liquid digestate used in this study was high in NH_4_^+^; the presence of K^+^ did not interfere with removing NH_4_^+^. Otherwise, potassium is an essential macronutrient and is most abundantly absorbed cation playing an important role in algae growth.

The initial NH_4_^+^:Mg^2+^:PO_4_^3−^ molar ratio and pH of the reaction system significantly affected the precipitation results [12], especially with respect to the remaining N and P in the struvite-precipitated supernatant, which determined the NH_4_^+^-N levels and N:P mass ratio in the supernatant. High NH_4_^+^-N may inhibit microalgal growth, but inhibitory thresholds vary widely within microalgal species [9]. In general, when the NH_4_^+^-N concentrations are below 100 mg/l, the growth of most microalgae will not be significantly inhibited [11, 34]. Only at a NH_4_^+^:Mg^2+^:PO_4_^3−^ ratio of 1:1:0.95, and a pH 8.0 and 8.5, did the remaining NH_4_^+^-N in the supernatant exceed 100 mg/l (Table 2).

**Table 2:** Characteristics of struvite-precipitated supernatant under different NH_4_^+^:Mg^2+^:PO_4_^3−^ molar ratio and pH

NH_4_^+^:Mg^2+^:PO_4_^3−^ molar ratio	pH	NH_4_^+^-N (mg/l)	NO_3_^−^-N (mg/l)	PO_4_^3−^-P (mg/l)	N:P mass ratio	Transmittance (%)	Salinity (%)	NH_4_^+^-N removal rate (%)
1:1:0.95	8.0	142.52 ± 2.10^a^	0.692 ± 0.021^a^	35.47 ± 0.48^a^	4.12 ± 0.68^a^	70.00 ± 0.76^a^	0.72 ± 0.21^a^	81.18 ± 0.37^a^
	8.5	118.71 ± 0.48^b^	0.771 ± 0.049^a^	29.87 ± 0.32^b^	4.05 ± 0.22^a^	71.12 ± 0.66^a^	0.69 ± 0.11^b^	83.33 ± 0.08^b^
	9.0	78.06 ± 0.25^c^	0.755 ± 0.074^a^	26.24 ± 0.70^c^	3.06 ± 0.42^b^	71.78 ± 0.74^a^	0.70 ± 0.01^ab^	86.41 ± 0.04^c^
1:1:1	8.0	90.07 ± 0.08^d^	0.030 ± 0.019^b^	34.12 ± 0.39^d^	2.70 ± 0.73^b^	74.65 ± 1.33^b^	0.67 ± 0.07^b^	85.32 ± 0.01^d^
	8.5	83.78 ± 0.82^e^	0.092 ± 0.076^b^	22.78 ± 0.51^e^	3.75 ± 0.04^b^	77.36 ± 1.34^b^	0.61 ± 0.08^b^	86.41 ± 0.14^c^
	9.0	66.56 ± 0.52^f^	0.063 ± 0.048^b^	16.36 ± 0.07^f^	4.16 ± 0.23^a^	74.82 ± 0.86^b^	0.62 ± 0.08^b^	88.41 ± 0.09^e^
1:1.2:1.2	8.0	71.08 ± 1.38 ^g^	0.053 ± 0.032^b^	18.92 ± 1.52 ^g^	3.86 ± 0.57^b^	72.19 ± 1.53^a^	0.64 ± 0.03^b^	89.62 ± 0.24^f^
	8.5	57.85 ± 0.66 ^h^	0.046 ± 0.042^b^	8.51 ± 0.84 ^h^	7.04 ± 0.72^c^	78.28 ± 0.38^bc^	0.66 ± 0.03^b^	91.63 ± 0.11 ^g^
	9.0	38.95 ± 0.74^i^	0.071 ± 0.016^b^	7.02 ± 1.21 ^h^	5.65 ± 0.43^d^	79.60 ± 0.01^d^	0.67 ± 0.02^b^	93.22 ± 0.13 ^h^

Results are expressed as the mean ± SD (*n* = 3)

^a,b,c^Different letters in the same row indicate significant differences at *p* < 0.05

The initial NH_4_^+^-N concentration of the liquid digestate used in this study was 591.2 mg/l. As more PO_4_^3−^ and Mg^2+^ was added, less NH_4_^+^-N remained. In addition, at the same NH_4_^+^:Mg^2+^:PO_4_^3−^ molar ratio, the remaining NH_4_^+^-N concentration decreased with an increasing pH value. At a NH_4_^+^:Mg^2+^:PO_4_^3−^ ratio of 1:1.2:1.2 and pH of 9.0, the NH_4_^+^-N concentration in supernatant was as low as 38.95 mg/l, indicating a NH_4_^+^ removal rate of more than 90% (Table 2). This led to the hypothesis that this method could be used to treat liquid digestates containing up to 1000 mg/l of NH_4_^+^-N, to meet the 100-mg/l NH_4_^+^-N concentration requirements for microalgal growth.

The N:P ratio is another important factor affecting microalgae growth. According to the typical microalgae composition formula (C_106_H_181_O_45_N_16_P), optimal microalgae growth occurs when the mass ratio of N to P that can be absorbed by microalgae is approximately 7:1 [17]. NH_4_^+^-N, NO_3_^−^-N, and PO_4_^3−^-P are the main forms of N and P absorbed by microalgae [35]; as such, the ratio of the sum of NH_4_^+^-N and NO_3_^−^-N to PO_4_^3−^-P was used to evaluate the N:P ratio in this study. The N:P mass ratio in the supernatants ranged from 2.70 to 7.04 (Table 2). At a NH_4_^+^:Mg^2+^:PO_4_^3−^ molar ratio of 1:1.2:1.2 and a pH of 8.5, the N:P mass ratio in the supernatant was 7.04, approaching the ideal value. Moreover, at this condition, the supernatant had the second highest transmittance and NH_4_^+^ removal rate.

**Fig. 3:**
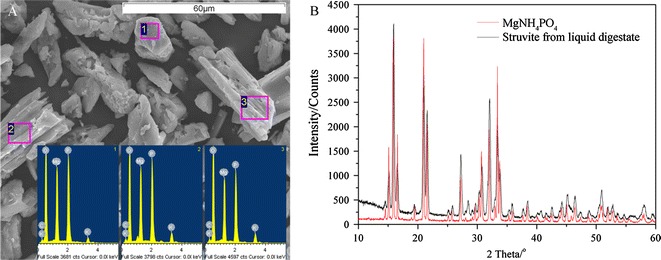
Scanning electron microscopy with energy dispersive analysis (**a**) and X-ray diffraction patterns (**b**) of struvite from liquid digestate (NH_4_^+^:Mg^2+^:PO_4_^3−^ molar ratios at 1:1.2:1.2, the stirring was stopped as soon as the pH reached to 8.5)

The shape and composition of the precipitates formed at a NH_4_^+^:Mg^2+^:PO_4_^3−^ molar ratio of 1:1.2:1.2 and a pH of 8.5 were further analyzed using SEM–EDS and XRD techniques. The precipitates showed a typical orthorhombic crystal shape and surface characterization (Fig. 3A), similar to results obtained from other pig slurry and described by Cerrillo et al. [18]. The crystal surface linked some ‘amorphous’ materials and contained a trace level of carbon (C). This probably related to the suspended solids generated from the liquid digestate. The XRD patterns also support the findings that the precipitates were made up of a mixture of struvite and amorphous materials. The prominent peaks of the precipitate matched the standard model for struvite very well, but there was an uneven baseline induced by the amorphous material (Fig. 3B). Flocculating suspended solids is one of the reasons that struvite precipitation improves the transmittance.

In summary, to achieve optimal transmittance, NH_4_^+^-N levels and N:P mass ratio for microalgal growth, the NH_4_^+^:Mg^2+^:PO_4_^3−^ ratio of the liquid digestate should first be adjusted to 1:1.2:1.2 using KH_2_PO_4_ and MgCl_2_ with continuous stirring. The pH should then be adjusted to 8.5 by adding NaOH. At that point, the stirring should be stopped. The supernatant obtained under this condition was used for the subsequent microalgae culture.

### Screening of suitable algal species

Nine microalgal species were cultured in the struvite-precipitated supernatant (Table 3). After 7 days of cultivation, *D. ehrenbergianum* showed the highest specific growth rate, followed by *C. regularis* FACHB-1068 and *S. obliquus* FACHB-417. In contrast, *C. pyrenoidosa* FACHB-9, *B. braunii* FACHB-357, *H. pluvialis* FACHB-712, and *S. platensis* FACHB-900 showed almost no growth during this period.

**Table 3:** Specific growth rate (μ) of different microalgae strains cultured in struvite-precipitated supernatant

Species	Specific growth rate
*Chlorella regularis* FACHB-1068	0.097 ± 0.016^a^
*C. pyrenoidosa* FACHB-9	–
*Botryococcus braunii* FACHB-357	–
*Scenedesmus obliquus* FACHB-417	0.089 ± 0.026^a^
*Dictyosphaerium ehrenbergianum* FACHB-1223	0.144 ± 0.025^b^
*Haematococcus pluvialis* FACHB-712	–
*Spirulina subsalsa* FACHB-351	0.069 ± 0.031^c^
*S. platensis* FACHB-900	–
*S. maxima* FACHB-438	0.084 ± 0.015^a^

Results are expressed as the mean ± SD (*n* = 3)

^a,b,c^Different letters in the same row indicate significant differences at *p* < 0.05

The genus *Dictyosphaerium* is found in both marine and fresh water environments [36] and some species in this genus have a strong ability to adapt to extreme environments [37]. For example, *D. chlorelloides* can survive in alkaline and moderately acidified aquatic environments containing hexavalent chromium [38] and *Dictyosphaerium* sp. has been found in high rate algal ponds (HRAPs) used for wastewater treatment [39]. *D. ehrenbergianum* was designated as a type species of the genus of *Dictyosphaerium* [37]. To date, research on this species has mainly focused on its taxonomy [40, 41]; therefore, this study provides a novel application for this alga.

### Growth of *D. ehrenbergianum* in struvite-precipitated supernatant

**Fig. 4:**
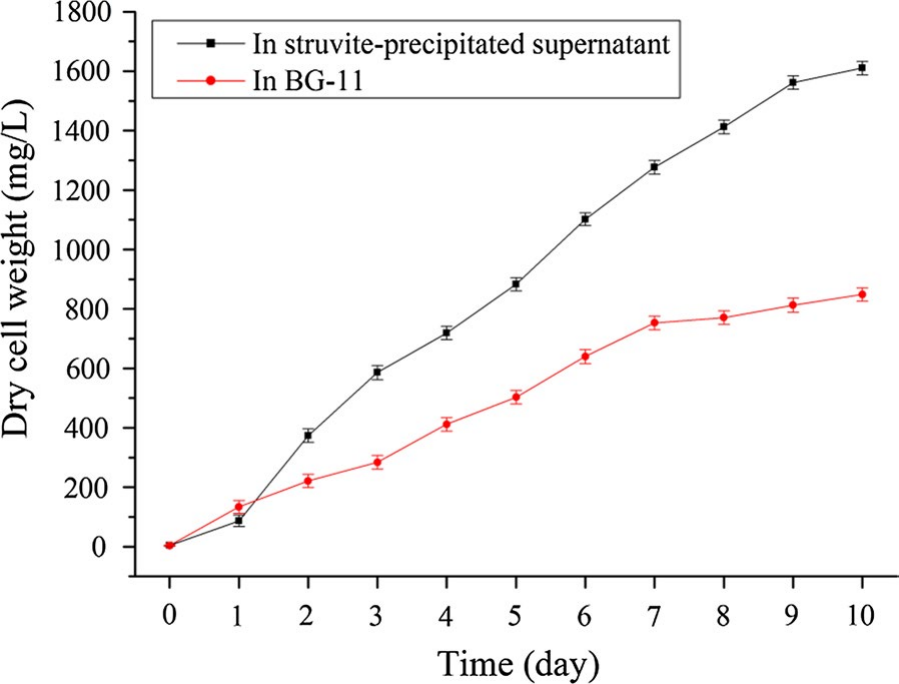
Growth curve of *Dictyosphaerium ehrenbergianum* in struvite-precipitated supernatant and BG-11. Errors bars show standard deviation (*n* = 3)

The growth curve of *D. ehrenbergianum* based on DCW was monitored in both the struvite-precipitated supernatant and BG-11 (Fig. 4). After a 1-day adaptation period, *D. ehrenbergianum* showed a higher growth rate in the supernatant than in BG11. By the seventh day, the growth of *D. ehrenbergianum* cultured in BG-11 stagnated; however, *D. ehrenbergianum* continued to grow in the supernatant. BG-11 medium, designed to cultivate blue–green algae, is now widely used to grow many microalgal strains, including the genus *Dictyosphaerium* [23, 38]; however, our results indicate that the struvite-precipitated supernatant was more conducive to *D. ehrenbergianum* biomass accumulation.

Table 4 shows the biomass and lipid productivity of *D. ehrenbergianum* in both the supernatant and BG-11 after 10 days of cultivation in the same culture conditions. The microalgae exhibited higher biomass productivity (161.06 mg/l/days) in the supernatant than in the BG-11 media. However, the biomass productivity is still lower than reported in other studies [42]. This may be because of the low light intensity of the light incubator (about 40–50 μmol/m^2^/s) used for this study; similar results were also found in *Chlorella* sp. [43].

**Table 4:** Biomass productivity, lipid content, lipid productivity, and fatty acid profiles of *Dictyosphaerium ehrenbergianum* in struvite-precipitated supernatant and BG-11

	In struvite-precipitated supernatant	In BG-11
Biomass productivity (mg/l/days)	161.06 ± 3.71	84.87 ± 6.24
Lipid content (% DW)	34.33 ± 1.52	23.67 ± 1.15
Lipid productivity (mg/l/days)	55.29 ± 2.37	20.09 ± 1.83
Fatty acids (% of total fatty acids)		
C16:0	21.09 ± 1.04	17.46 ± 0.62
C18:0	3.27 ± 0.16	1.32 ± 0.33
C16:1	7.43 ± 0.13	2.41 ± 0.47
C18:1	13.48 ± 0.63	11.05 ± 0.59
C16:2	7.05 ± 0.75	8.60 ± 0.50
C16:3	9.46 ± 0.60	15.42 ± 1.21
C18:2	17.76 ± 0.73	20.23 ± 1.21
C18:3	19.37 ± 0.73	22.24 ± 1.09

In this study, the lipid level of *D. ehrenbergianum* cultured in BG-11 was 23.67%, which is close to the level found for *Dictyosphaerium* CFR 5-01/FW by Vidyashankar et al. [44]. However, the lipid content of *D. ehrenbergianum* cultured in the struvite-precipitated supernatant was as high as 34.33%, resulting in lipid productivity of 55.29 mg/l/days. This indicates that the supernatant was also beneficial for *D. ehrenbergianum* lipid accumulation. Other research has shown that lipid accumulation can be promoted when microalgal cells are cultivated in stressed conditions, such as high salinity [45]. By adding KH_2_PO_4_ and MgCl_2_, the presence of K^+^, and other ions, increased the salinity (from 0.26 to 0.66%) of the supernatant following struvite precipitation; therefore, the increased salinity in the supernatant did not inhibit microalgae growth, but actually contributed to greater lipid accumulation.

The predominant fatty acids of *D. ehrenbergianum* cultured in both the struvite-precipitated supernatant and BG-11 were C16 and C18 (Table 4), which are the main components of biodiesels [10]. This indicates that the oil from *D. ehrenbergianum* is an ideal biodiesel alternative. The microalgae biodiesels usually have poor oxidative stability due to the low monounsaturated fatty acid (MUFA) and saturated fatty acid (SaFA) content [46]. The proportion of total MUFAs and SaFAs of *D. ehrenbergianum* increased from 32.24% in BG-11 to 45.27% in the supernatant, whereas the proportion of polyunsaturated fatty acid (PUFA) was decreased. This indicated that the lipid profiles of *D. ehrenbergianum* could change with growth conditions, the supernatant generated through in this study was more favorable for culturing *D. ehrenbergianum* for ideal biodiesel production. Similar results were also found in the previous study [47].

## Conclusions

This study constructed a new way to combine liquid digestate treatment and microalgal cultivation. The study also determined the optimal struvite precipitation conditions for pretreating liquid digestate intended for use as a microalgal culture medium. KH_2_PO_4_ and MgCl_2_ were the optimum source of Mg^2+^ and PO_4_^3−^ to adjust the NH_4_^+^:Mg^2+^:PO_4_^3−^ molar ratio to 1:1.2:1.2. Continued stirring should be stopped when the pH reaches 8.5. *D. ehrenbergianum* grew best in the struvite-precipitated supernatant, which enhanced *D. ehrenbergianum* biomass productivity and lipid content, and also improved the accumulation of MUFAs and SaFAs. The struvite-precipitated liquid digestate can be used to culture *D. ehrenbergianum* for biodiesel production.

## References

[1] Wijffels RH, Barbosa MJ (2010). An outlook on microalgal biofuels. Science.

[2] Williams PJL, Laurens LML (2010). Microalgae as biodiesel & biomass feedstocks: review & analysis of the biochemistry, energetics & economics. Energ Environ Sci.

[3] Zhu L (2015). Microalgal culture strategies for biofuel production: a review. Biofuels Bioprod Bioref..

[4] Delrue F, Alvarez-Diaz PD, Fon-Sing S, Fleury G, Sassi JF (2016). The environmental biorefinery: using microalgae to remediate wastewater, a win–win paradigm. Energies..

[5] Chiu SY, Kao CY, Chen TY, Chang YB, Kuo CM, Lin CS (2014). Cultivation of microalgal Chlorella for biomass and lipid production using wastewater as nutrient resource. Bioresour Technol.

[6] Monlau F, Sambusiti C, Ficara E, Aboulkas A, Barakat A, Carrere H (2015). New opportunities for agricultural digestate valorization: current situation and perspectives. Energ Environ Sci..

[7] Nkoa R (2013). Agricultural benefits and environmental risks of soil fertilization with anaerobic digestates: a review. Agron Sustain Dev.

[8] Fuchs W, Drosg B (2013). Assessment of the state of the art of technologies for the processing of digestate residue from anaerobic digesters. Water Sci Technol.

[9] Xia A, Murphy JD (2016). Microalgal cultivation in treating liquid digestate from biogas systems. Trends Biotechnol.

[10] Wang L, Li Y, Chen P, Min M, Chen Y, Zhu J (2010). Anaerobic digested dairy manure as a nutrient supplement for cultivation of oil-rich green microalgae *Chlorella* sp. Bioresour Technol.

[11] Uggetti E, Sialve B, Latrille E, Steyer JP (2014). Anaerobic digestate as substrate for microalgae culture: the role of ammonium concentration on the microalgae productivity. Bioresour Technol.

[12] Darwish M, Aris A, Puteh MH, Abideen MZ, Othman MN (2015). Ammonium–nitrogen recovery from wastewater by struvite crystallization technology. Sep Purif Rev..

[13] Talboys PJ, Heppell J, Roose T, Healey JR, Jones DL, Withers PJ (2016). Struvite: a slow-release fertiliser for sustainable phosphorus management. Plant Soil.

[14] Estevez MM, Linjordet R, Horn SJ, Morken J (2014). Improving nutrient fixation and dry matter content of an ammonium-rich anaerobic digestion effluent by struvite formation and clay adsorption. Water Sci Technol.

[15] Cheng J, Xu J, Huang Y, Li Y, Zhou J, Cen K (2015). Growth optimisation of microalga mutant at high CO(2) concentration to purify undiluted anaerobic digestion effluent of swine manure. Bioresour Technol.

[16] Nelson NO, Mikkelsen RL, Hesterberg DL (2003). Struvite precipitation in anaerobic swine lagoon liquid: effect of pH and Mg:P ratio and determination of rate constant. Bioresour Technol.

[17] Karapinar Kapdan I, Aslan S (2008). Application of the Stover–Kincannon kinetic model to nitrogen removal by *Chlorella vulgaris* in a continuously operated immobilized photobioreactor system. J Chem Technol Biotechnol.

[18] Cerrillo M, Palatsi J, Comas J, Vicens J, Bonmatí A (2015). Struvite precipitation as a technology to be integrated in a manure anaerobic digestion treatment plant—removal efficiency, crystal characterization and agricultural assessment. J Chem Technol Biotechnol.

[19] Perera PWA, Han ZY, Chen YX, Wu WX (2007). Recovery of nitrogen and phosphorous as struvite from swine waste biogas digester effluent. Biomed Environ Sci.

[20] Khatoon H, Abdu Rahman N, Banerjee S, Harun N, Suleiman SS, Zakaria NH (2014). Effects of different salinities and pH on the growth and proximate composition of *Nannochloropsis* sp. and *Tetraselmis* sp. isolated from South China Sea cultured under control and natural condition. Int Biodeter BiodegrI..

[21] Zhang DM, Chen YX, Jilani G, Wu WX, Liu WL, Han ZY (2012). Optimization of struvite crystallization protocol for pretreating the swine wastewater and its impact on subsequent anaerobic biodegradation of pollutants. Bioresour Technol.

[22] Aiba S, Ogawa T (1977). Assessment of growth yield of a blue–green alga: spirulina platensis, in axenic and continuous culture. J General Microbiol..

[23] Stanier RY, Kunisawa R, Mandel M, Cohen-Bazire G (1971). Purification and properties of unicellular blue–green algae (Order *Chroococcales*). Bacteriol Rev..

[24] Song M, Pei H, Hu W, Ma G (2013). Evaluation of the potential of 10 microalgal strains for biodiesel production. Bioresour Technol.

[25] Santana H, Cereijo CR, Teles VC, Nascimento RC, Fernandes MS, Brunale P (2017). Microalgae cultivation in sugarcane vinasse: selection, growth and biochemical characterization. Bioresour Technol.

[26] Association APHA (2005). Standard methods for the examination of water and wastewater.

[27] Bligh EGDWJ (1959). A rapid method of total lipid extraction and purification. Can J Chem.

[28] Indarti E, Majid MIA, Hashim R, Chong A (2005). Direct FAME synthesis for rapid total lipid analysis from fish oil and cod liver oil. J Food Compos Anal.

[29] Kirst GO (1989). Salinity tolerance of eukaryotic marine algae. Annu Rev Plant Physiol Plant Mol Biol.

[30] Hwang JH, Church J, Lee SJ, Park J, Lee WH (2016). Use of microalgae for advanced wastewater treatment and sustainable bioenergy generation. Environ Eng Sci.

[31] Wilsenach JA, Schuurbiers CAH, van Loosdrecht MCM (2007). Phosphate and potassium recovery from source separated urine through struvite precipitation. Water Res.

[32] Yetilmezsoy K, Sapci-Zengin Z (2009). Recovery of ammonium nitrogen from the effluent of uasb treating poultry manure wastewater by map precipitation as a slow release fertilizer. J Hazard Mater.

[33] Martí N, Pastor L, Bouzas A (2010). Phosphorus recovery by struvite crystallization in WWTPs: influence of the sludge treatment line operation. Water Res.

[34] Park J, Jin HF, Lim BR, Park KY, Lee K (2010). Ammonia removal from anaerobic digestion effluent of livestock waste using green alga *Scenedesmus* sp. Bioresour Technol.

[35] Richmond A, Hu Q (2013). Handbook of microalgal culture: biotechnology and applied phycology.

[36] Guiry MD, Guiry GM. AlgaeBase. World-wide electronic publication. Galway: National University of Ireland; 2016. http://www.algaebase.org. Accessed 27 Dec 2016.

[37] Lopez-Rodas V, Marva F, Rouco M, Costas E, Flores-Moya A (2008). Adaptation of the chlorophycean *Dictyosphaerium chlorelloides* to stressful acidic, mine metal-rich waters as result of pre-selective mutations. Chemosphere.

[38] Pereira M, Bartolome MC, Sanchez-Fortun S (2013). Influence of pH on the survival of *Dictyosphaerium chlorelloides* populations living in aquatic environments highly contaminated with chromium. Ecotoxicol Environ Saf.

[39] Park JB, Craggs RJ, Shilton AN (2013). Enhancing biomass energy yield from pilot-scale high rate algal ponds with recycling. Water Res.

[40] Bock C, Pröschold T, Krienitz L (2011). Updating the genus *Dictyosphaerium* and description of Mucidosphaerium gen. nov (Trebouxiophyceae) based on morphological and molecular data. J Phycol.

[41] Zou S, Fei C, Song J, Bao Y, He M, Wang C (2016). Combining and comparing coalescent, distance and character-based approaches for barcoding microalgaes: a test with chlorella-like species (Chlorophyta). PLoS ONE.

[42] Cho DH, Ramanan R, Heo J, Shin DS, Oh HM, Kim HS (2016). Influence of limiting factors on biomass and lipid productivities of axenic *Chlorella vulgaris* in photobioreactor under chemostat cultivation. Bioresour Technol.

[43] Zhou X, Ge H, Xia L, Zhang D, Hu C (2013). Evaluation of oil-producing algae as potential biodiesel feedstock. Bioresour Technol.

[44] Vidyashankar S, VenuGopal KS, Swarnalatha GV, Kavitha MD, Chauhan VS, Ravi R (2015). Characterization of fatty acids and hydrocarbons of chlorophycean microalgae towards their use as biofuel source. Biomass Bioenerg.

[45] Markou G, Nerantzis E (2013). Microalgae for high-value compounds and biofuels production: a review with focus on cultivation under stress conditions. Biotechnol Adv.

[46] Stansell GR, Gray VM, Sym SD (2011). Microalgal fatty acid composition: implications for biodiesel quality. J Appl Phycol.

[47] Olmstead ILD, Hill DRA, Dias DA (2013). A quantitative analysis of microalgal lipids for optimization of biodiesel and omega-3 production. Biotechnol Bioeng.

